# Editorial: Sprouts, microgreens and edible flowers: Modulation of quality in functional specialty crops

**DOI:** 10.3389/fpls.2022.1033236

**Published:** 2022-09-21

**Authors:** Marios C. Kyriacou, Andreas W. Ebert, Giedrė Samuolienė, Aušra Brazaitytė

**Affiliations:** ^1^ Department of Vegetable Crops, Agricultural Research Institute, Nicosia, Cyprus; ^2^ Genetic Resources and Seed Unit, World Vegetable Center, Tainan City, Taiwan; ^3^ Lithuanian Research Centre for Agriculture and Forestry, Institute of Horticulture, Babtai, Lithuania

**Keywords:** specialty crops, sensory and functional quality, phytochemical contents, bioactive compounds, healthy diets, growth stages, light intensity and quality, LED – light emitting diode

Sprouts, microgreens, and edible flowers constitute upcoming specialty crops increasingly esteemed for their sensory contribution to global gastronomy and their bioactive composition that potentially enhances human health. The expanding consumption of these crops is driven by their outstanding organoleptic characteristics, low fat content and rich bioactive composition comprising flavonoids, carotenoids, glucosinolates, vitamins, amino acids and minerals (Kyriacou et al., 2016).

The production of sprouts and microgreens involves significant physiological events associated with the mobilization of seed reserves, which fuel the ontogeny of seedlings and deliver more readily digestible forms of food for human consumption. Species selection, fertilization-biofortification, stress, light intensity, photoperiod, wavelength and growth stage impact microgreens physiology and, ultimately, their sensory and functional quality. Genotype selection, substrate properties and especially light conditions were the key factors appraised in the present Research Topic with respect to crop physiology, product quality, phytochemical composition and nutritional value. The advancement of light-emitting diode (LED) technology has rendered the use of supplemental lighting for indoor plant growth modules more feasible and energy-efficient (Alrifai et al., 2019). The LED technology facilitates the optimal tuning of spectral quality (wavelength), intensity (photon flux) and photoperiod that might elicit the activation of selective photoreceptors and enhance the phytochemical content of sprouts and microgreens (Bian et al., 2015; Brazaitytė et al., 2015).

While a plethora of works has described the effects of mostly far-red, red, blue, or ultraviolet photoreceptors on plant ontogeny and composition (Galvão and Fankhauser, 2015), information on other supplementary bandwidths remains limited. Samuoliené et al. examined the effect of LED green (520 nm), yellow (595 nm) and orange (622 nm) bandwidths, supplemental to the primary spectrum (blue - 447 nm, red - 638 and 665 nm, and far-red - 731 nm), on the secondary metabolism of brassicaceous microgreens. Yellow light elicited the increase of soluble carbohydrates in mizuna and broccoli microgreens, while all supplemental light components enhanced β-carotene content in mizuna and ascorbic acid content in kohlrabi. Orange light was found to improve mineral (Fe, Mg, and Ca) concentration in all genotypes. The metabolic changes observed in treated microgreens support the use of selected supplemental LED wavelengths in a genotype-specific mode to improve nutritionally valuable components in brassicaceous microgreens.

The genotype-specific modulatory effects of select spectral bandwidths were further examined by Kyriacou et al. as a tool for designing future crop-specific spectral management systems to enhance the phytochemical composition of microgreens. Dichromatic blue-red light was found to modulate growth-related parameters dependent on primary metabolism and to promote nitrate and nitrite assimilation. Conversely, monochromatic blue and red lights promoted nitrate accumulation in microgreens but also elicited a genotype-specific increase of lutein and β-carotene. Notwithstanding substantial genotypic differences, Orbitrap LC-MS/MS analysis revealed a trend across species for a decrease of specific polyphenolic constituents, particularly flavonol glycosides, as well as total polyphenols under blue-red light. As this work highlighted, combining select genetic background with appropriate light management may foster the production of microgreens with improved bioactive composition.

Light conditions are also of prime interest with respect to the growth and phytochemical composition of sprouts. In this respect, Chen et al. examined Chinese kale (*Brassica alboglabra*) sprouts response to increasing photoperiod (0, 8, 12 and 16 h/24 h, rest dark) under white or combined red-and-blue (RB) light sources in order to assess growth and glucosinolates content. Growth and dry matter content were highest in sprouts grown under 16-h RB light. Moreover, increasing the red/blue ratio in favor of blue light enhanced the accumulation of glucosinolates. Intriguingly, glucosinolate biosynthesis transcripts were more abundant in sprouts grown under red rather than blue light. Considering the differential expression of β-glucosidase family homolog genes related to glucosinolates degradation with respect to the red/blue ratio, it was concluded that degradation is implicated in the homeostasis of gucosinolates in Chinese kale sprouts.

Wang et al. examined the phytohormonal role of salicylic acid (SA) and the effect of blue light on the accumulation of indole-3-carbinol (I3C), a secondary metabolite with strong anti-cancer capacity present in cruciferous plants, including broccoli (*Brassica oleracea* L.). They observed that blue light promoted the accumulation of I3C in broccoli sprouts, whereas exogenous SA treatment inhibited its accumulation. RNA sequence and pathway analysis indicated that blue light downregulated the expression of genes related to SA biosynthesis and upregulated the expression of I3C genes, suggesting that SA interferes negatively with I3C accumulation in broccoli sprouts treated with blue light.

The impact of altitude on the nutritive and phytochemical profile and the antioxidant capacity of mungbean and lentil genotypes grown as microgreens was examined by Priti et al.. Significant genotypic variation was identified with respect to ascorbic acid, tocopherol, carotenoids, flavonoids, total phenolics, *in vitro* antioxidant capacity, peroxide activity, protein content, peroxidase and catalase enzymes, micronutrient and macronutrient content. However, most of the studied parameters were found superior in high-altitude microgreens, which was attributed to the wider day-night temperature amplitude, the photosynthetically active radiation (PAR) and the UV-B levels. Furthermore, microgreens of both species abounded in K, P and Ca. These characteristics render Fabaceae microgreens a valuable asset for food security of communities at higher altitudes.

Finally, D’Imperio et al. examined how the nutritional quality of Mizuna and Rapini microgreens is impacted by the incorporation of *Posidonia oceanica* (L.) Delile seagrass residues in a commercial peat-based growth substrate at variable rates. *Posidonia* residues increased the I and B content of microgreens. Estimation of consumer safety, daily intake, percentage of recommended daily allowance for I and hazard quotient (HQ) for I intake through consumption of Rapini microgreens confirmed the possibility of improving microgreens’ nutritional profile by incorporating this sustainable natural material as a growth substrate component.

The niche in the horticultural supply chain for these specialty crops will undoubtedly benefit from future scientific output on their response to preharvest and postharvest applications that can enhance their sensory and functional profile. The current compilation of high-standard scientific papers may serve as the springboard for such work.

## Author contributions

Conceptualization, MK, AE, GS, AB; investigation, MK, AE, GS, AB; writing—review and editing MK, AE, GS, AB; All authors have read and agreed to the published version of the manuscript.

## Conflict of interest

The authors declare that the research was conducted in the absence of any commercial or financial relationships that could be construed as a potential conflict of interest.

## Publisher’s note

All claims expressed in this article are solely those of the authors and do not necessarily represent those of their affiliated organizations, or those of the publisher, the editors and the reviewers. Any product that may be evaluated in this article, or claim that may be made by its manufacturer, is not guaranteed or endorsed by the publisher.
